# Expression of IMP1 Enhances Production of Murine Leukemia Virus Vector by Facilitating Viral Genomic RNA Packaging

**DOI:** 10.1371/journal.pone.0015881

**Published:** 2010-12-29

**Authors:** Yun Mai, Guangxia Gao

**Affiliations:** Key Laboratory of Infection and Immunity, Institute of Biophysics, Chinese Academy of Sciences, Beijing, China; The University of Hong Kong, Hong Kong

## Abstract

Murine leukemia virus (MLV)-based retroviral vector is widely used for gene transfer. Efficient packaging of the genomic RNA is critical for production of high-titer virus. Here, we report that expression of the insulin-like growth factor II mRNA binding protein 1 (IMP1) enhanced the production of infectious MLV vector. Overexpression of IMP1 increased the stability of viral genomic RNA in virus producer cells and packaging of the RNA into progeny virus in a dose-dependent manner. Downregulation of IMP1 in virus producer cells resulted in reduced production of the retroviral vector. These results indicate that IMP1 plays a role in regulating the packaging of MLV genomic RNA and can be used for improving production of retroviral vectors.

## Introduction

Murine leukemia virus (MLV)-based retroviral vector is widely used for gene transfer in clinical applications and basic research. The most commonly encountered problem is that the virus titer is too low. Retroviral vectors produced by DNA transfection of producing cells usually contain a large amount of non-infectious virus. The ratio of infectious virions to the total MLV particles could be as low as 1/100 [1]. The large amount of non-functional virion particles not only makes it cumbersome and costly to increase the titers of retroviral vectors for gene therapy but also produces a noise for virology research. Inactivation of progeny viruses can be attributed to abrogation or impairment of viral structural proteins, enzymes or viral genomic RNAs [2]–[6]. Great efforts have been made to increase the proportion of functional retroviral vectors. The strategies include optimizing the recipe of culture medium [7], [8], cultivation temperature [9] and feeding schedule of virus producer cells [10]–[13], improving the packaging cell lines [14], [15], modifying the stoichiometric proportion of viral proteins and viral genomic RNAs [16], [17], and removing the non-infectious particles[18].

In vitro experiments suggested that host cells control the production of genomic RNA-deficient virus. The synthesis of such deficient virus was greatly different in different host cells [2], suggesting that some cellular factors may be involved in determining the viral genomic RNA packaging efficiency. It also suggests that it is possible to improve viral genomic RNA packaging and thereby infectivity of retroviral vectors by manipulating the expression of some cellular factors in the producer cells.

For the wild type replication-competent MLV virus, the genome-length viral RNAs of MLV are believed to be separated into two non-equilibrating pools in virus producer cells, with one serving as mRNA for translation of Gag and Gag/Pol polyproteins and the other as genomic RNA for packaging [19]–[25]. Studies addressing the preference of homodimerization of MLV viral genomic RNAs suggest that the fates of the unspliced viral RNAs as mRNAs or genomic RNAs are determined immediately after transcription and that these two populations still can exchange before genomic RNA dimer maturation [25]. Since Gag alone is able to form virus like particles, some of the progeny viruses are possibly empty when the levels of packagable genomic RNAs in the virus producer cells are low. For MLV vector production, the viral proteins are usually expressed separately and the viral genomic RNA is designed only for packaging. Thus, it is possible to increase the abundance of the genomic RNA in the packaging pool without affecting the expression of viral proteins and thus increase the production of infectious retroviral vectors.

Insulin-like growth factor II mRNA binding protein 1 (IMP1) is a member of the VICKZ family [26], which are highly conserved RNA-binding proteins involved in posttranscriptional regulation including RNA trafficking [27], stabilization [28], [29] and translation regulation [30], [31]. For example, IMP1, also known as the coding region determinant-binding protein (CRD-BP), protects *c-myc* mRNA from endonuclease cleavage and increases its stability [28], [29]. IMP1 has also been implicated in the regulation of the translation of certain mRNAs. IMP1 bound specifically to 5′ untranlated region of leader 3 mRNA of Insulin-like Growth Factor II (IGF II), but not leader 4 mRNA, and repressed its translation [30], [31].

Based on the abilities of IMP1 to inhibit the translation and to increase the stability of some RNAs, we investigated the role of IMP1 in regulating the production of infectious MLV vectors. Our results demonstrated that overexpression of IMP1 increased the viral genomic RNA levels both in virus producer cells and virions and thereby the production of functional MLV vector.

## Results

### Overexpression of IMP1 increased the production of MLV vector in a dose-dependent manner

**Figure 1:**
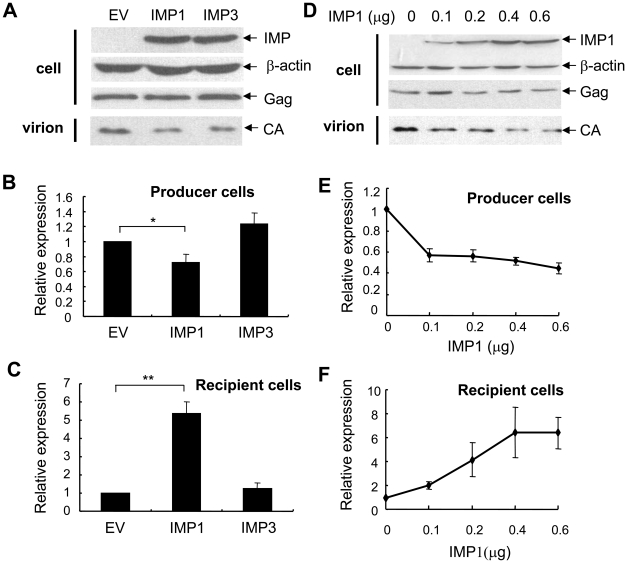
Overexpression of IMP1 in producer cells increased the production of infectious MLV vector in a dose-dependent manner. The MLV vector producing constructs pCMV-VSVG, pHIT60 and pMA-Luc in a total of 3.6 µg at the ratio of 1.3∶1.3∶1, and 0.01 µg of pRL-TK, a plasmid expressing renilla luciferase, were cotransfected into 293T cells in 10 cm dishes together with 0.4 µg of empty vector, pcDNA4-IMP1 or pcDNA4-IMP3 (A–C), or with the indicated amounts of IMP1 (D–F). At 48 h posttransfection, the virus was harvested to infect 293 cells and the luciferase activities in the producer cells were measured. At 48 h postinfection, the luciferase activity was measured in the recipient cells. (A) (D) The indicated proteins in the producer cells or virions were detected by Western blotting. (B) (E) Firefly luciferase activity normalized by reneilla luciferase activity in the producer cells. (C) (F) Firefly luciferase activity in the recipient cells normalized by reneilla luciferase activity in the producer cells. The data are means ± SD of three independent measurements. *denotes p<0.05, **denotes p<0.01.

To test whether expression of IMP1 affected the production of MLV-luc, a retroviral vector carrying the firefly luciferase reporter [32], [33], IMP1 was overexpressed in MLV-luc producing cells. The plasmid expressing IMP1 was transiently transfected into 293T cells together with MLV-luc producing constructs. A plasmid expressing reneilla luciferase was also included to serve as a control for transfection efficiency and sample handling. The viruses were used to infect 293 cells, and the luciferase activities in these cells were measured. The firefly luciferase activity normalized by the reneilla lucirferase activity was used as an indicator for virus production. IMP3, an IMP family member with high similarity to IMP1, was used for comparison. In the producer cells transfected with IMP1, the expression of viral Gag protein was little affected (Fig. 1A), and the normalized firefly luciferase activity was modestly lower compared with that in the cells transfected with empty vector or IMP3 (Fig. 1B). In contrast, the normalized firefly luciferase activity in the recipient cells infected with MLV-luc produced in the presence of IMP1 was much higher than that in the presence of empty vector or IMP3 (Fig. 1C). Both mouse IMP1 (mIMP1) and human IMP1 (hIMP1) affected MLV-luc production similarly (data not shown) and mIMP1 was used in later experiments.

To test whether IMP1 enhanced the production of infectious MLV-luc in a dose-dependent manner, different amounts of the IMP1-expressing plasmid was transfected with the MLV-luc producing constructs. Consistent with the above results, the expression levels of Gag protein in the producer cells were little changed (Fig. 1D), and the luciferase activity in the producer cells was reduced by about 50% (Fig. 1E) at high dosages. In contrast, IMP1 overexpression increased the luciferase activity in the recipient cells in an IMP1 dose-dependent manner (Fig. 1F). These results established that overexpression of IMP1 enhanced the production of infectious MLV-luc pseudovirus.

### IMP1 increased viral genomic RNA incorporation

**Figure 2:**
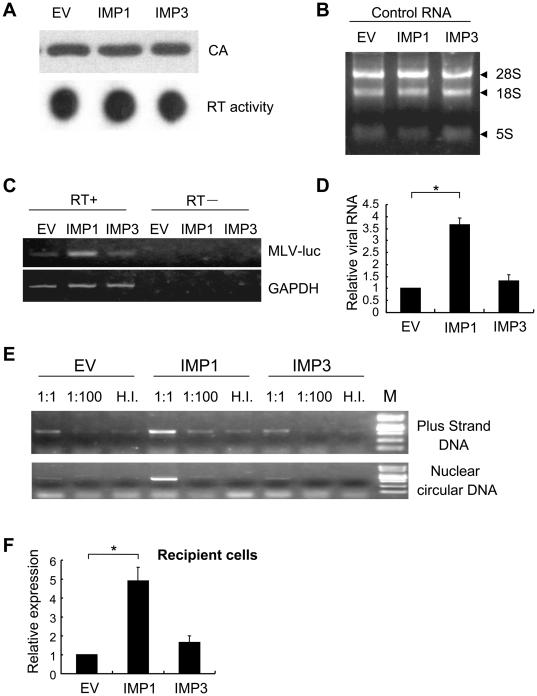
IMP1 increased viral genomic RNA packaging into virions. The MLV-luc producing constructs pCMV-VSVG, pHIT60 and pMA-Luc were cotransfected into 293T cells with empty vector, pcDNA4-IMP1 or pcDNA4-IMP3. At 48 h posttransfection, the virus was collected, purified by 25%/45% step gradient centrifugation and then concentrated by centrifugation through 25% sucrose cushion. Equal amounts of virions were used for further analyses, as judged by the levels of CA measured by Western blotting. The viral genomic RNA was isolated by Trizol extraction. To help sample handling, 10 µg of the total RNA of 293 cells was added to each virus sample before performing RNA-extraction. The samples were treated with DNase I to remove possible plasmid DNA contamination. The viral genomic RNA levels were measured by RT-PCR or quantitative RT-PCR (qPCR). The purified virus was also used to infect 293 cells to measure the infectivity. The Hirt DNA was extracted from the infected cells and the levels of viral DNA were measured by PCR. (A) The purified virions were resuspended. An aliquot was lysed by boiling in the sample buffer and subjected to Western blotting using anti-CA antibody (upper). Another aliquot was assayed for the RT activity by the homopolymer assay (lower). (B) The viral genomic RNA and the extrinsic total RNA of 293 cells were extracted. An aliquot of the RNA was subjected to electrophoresis followed by ethedium bromide staining. (C) (D) The RNA was reverse transcribed using oligo(dT)_18_ as the primer. The levels of the cDNA were analyzed by PCR (C) or qPCR (D). GAPDH from the added cellular RNA was used as an internal control. The qPCR data are means ± SD from three independent measurements. *denotes p<0.05. (E) The virus samples at the indicated dilution were used to infect 293 cells. At 12 h postinfection, Hirt DNA was extracted and the levels of the indicated viral DNAs were measured by PCR. HI: heat-inactivated virus. (F) An aliquot of the virus was used to infect 293 cells. At 48 h postinfection, the cells were lysed and the luciferase activity was measured. The data are means ± SD from three independent measurements. *denotes p<0.05.

To test whether IMP1 facilitated the incorporation of viral genomic RNA into the virions, the viral genomic RNA levels in the virion particles were measured. MLV-luc pseudovirus produced in the presence of empty vector, IMP1 or IMP3 were purified and concentrated. The same amounts of virion particles were used, as indicated by the amount of capsid (CA) protein in the virions (Fig. 2A, upper panel). The reverse transcriptase (RT) activity in the virions was first measured by the homopolymer RT assay and no difference was observed (Fig. 2A, lower panel), indicating that IMP1 overexpression did not increase the incorporation of RT into the virions. The viral genomic RNA was extracted from the virions. To help sample handling, total RNA of naïve 293 cells was added to each virus sample before performing RNA-extraction, which also served as an indicator for the quality of the RNA sample (Fig. 2B). The viral genomic RNA levels were first analyzed by reverse transcription followed by PCR. Indeed, the RNA level in the virions produced in the IMP1-expressing cells was significantly higher than that produced in the control cells (Fig. 2C). When measured by quantitative PCR, the RNA level in the virions produced in the IMP1-expressing cells was about 3.5-fold of that in the virions produced in the control cells (Fig. 2D). To confirm that the viral genomic RNA in the virions produced in the IMP1-expressing cells was functional, 293 cells were infected with the virus and the viral DNAs in the recipient cells were analyzed. As expected, higher level of viral DNA was detected in the cells infected with the virus produced in the IMP1-expressing cells (Fig. 2E). Consistent with the previous results (Fig. 1C), when the same samples of virus were used to infect 293 cells, the luciferase activity in the recipient cells infected with virus produced in IMP1-expressing cells was about 5-fold of that in the cells infected with virus produced in the control cells (Fig. 2F).

### IMP1 bound to and stabilized MLV-luc RNA in the virus producer cells

**Figure 3:**
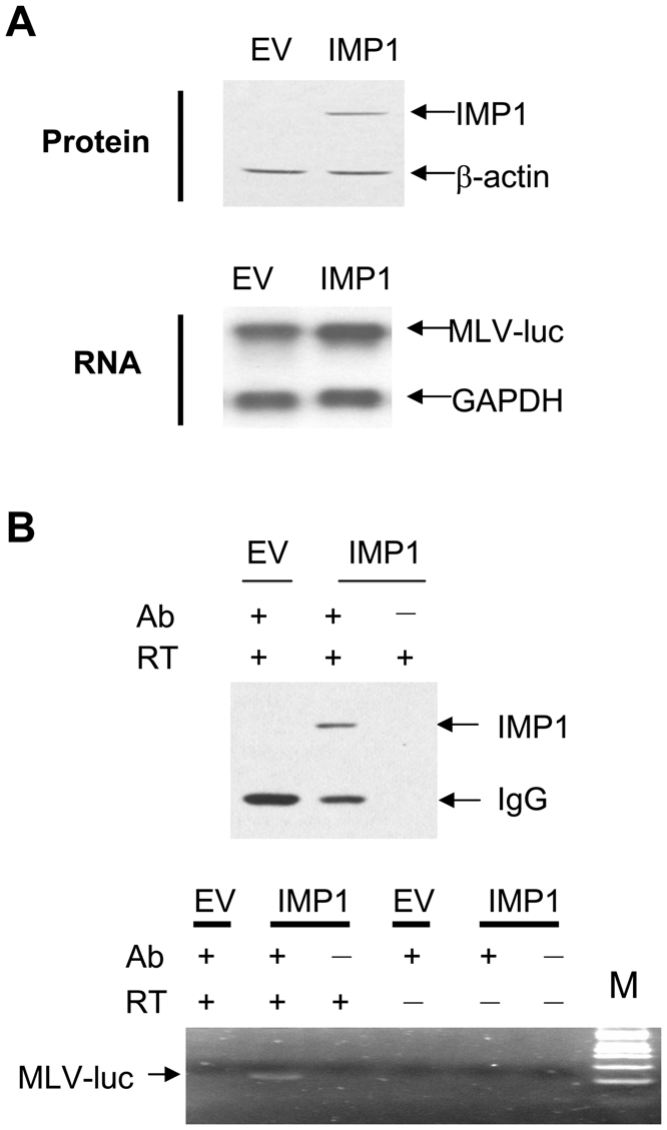
IMP1 bound to MLV-luc RNA. pMA-luc was transfected together with pcDNA4-IMP1 or empty vector into 293T cells in 6 cm dishes. (A) At 48 h posttransfection, the cells were harvested. The expression of IMP1 was examined by Western blotting (upper) and the expression of MLV-luc RNA was detected by Northern blotting (lower). (B) The cell lysates were immunoprecipitated with anti-myc antibody and Protein G beads. An aliquot of the beads was used to examine the immonuprecipitation of IMP1 by Western blotting (upper). The RNA bound to the beads was extracted and detected by RT-PCR (lower). Ab: anti-myc antibody; RT: reverse transcriptase.

The increased incorporation of MLV-luc RNA into the virion particles could be accounted for by increased abundance of the RNA in the producer cells or more efficient encapsidation of the RNA into the virions, or both. To test these possibilities, we first analyzed whether IMP1 bound to the viral genomic RNA in the producer cells. IMP1 was co-expressed with MLV-luc (Fig. 3A) in 293T cells. IMP1 was immunoprecipitated (Fig. 3B, upper panel) and the associated viral genomic RNA was detected by RT-PCR (Fig. 3B lower panel). Indeed, immunoprecipitation of IMP1 precipitated MLV-luc RNA (Fig. 3B). These results demonstrated that IMP1 bound MLV-luc RNA.

**Figure 4:**
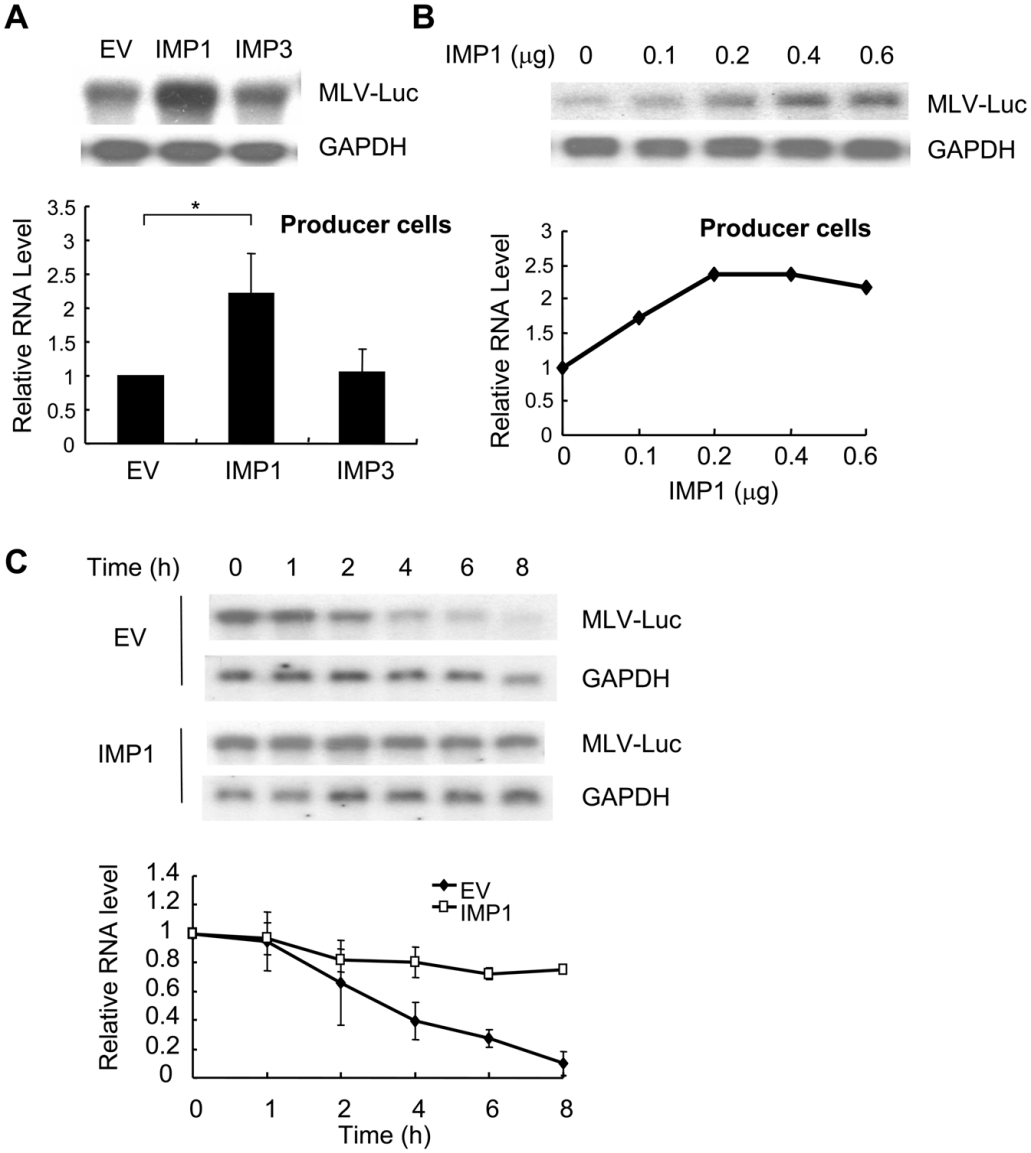
IMP1 stabilized the viral genomic RNA in the producer cells. (A) The MLV-luc vector producing constructs were cotransfected into 293T cells with empty vector, pcDNA4-IMP1 or pcDNA4-IMP3. At 48 h posttransfection, the total RNA was extracted and subjected to Northern blotting analysis (upper). The relative levels of the viral genomic RNA were measured using Phosphoimager and normalized by the GAPDH mRNA levels (lower). The data are means ± SD from three independent measurements. *denotes p<0.05. (B) The MLV vector producing constructs were cotransfected into 293T cells in 10 cm dishes together with the indicated amounts of pcDNA4-IMP1. At 48 h posttransfection, total RNA was extracted and subjected to Northern blotting analysis (upper). The relative levels of the viral RNA were measured using Phosphoimager and normalized by the GAPDH mRNA levels (lower). (C) pMA-Luc was cotransfected with empty vector or pcDNA4-IMP1 into 293T cells. At 24 h posttransfection, the cells were equally divided into 7 dishes and 12 h later actinomycin D was added to the media at a final concentration of 5 µg/ml. At the indicated time points, the cells were lysed and the RNA was extracted and subjected to Northern blotting analysis (upper). The relative levels of the viral RNA were measured using Phosphoimager and normalized by the GAPDH mRNA levels (lower). The data are means ± SD from two independent measurements.

To test whether expression of IMP1 increased the abundance of the viral genomic RNA in the producer cells, the viral genomic RNA levels in the producer cells were analyzed by Northern blotting. In the IMP1-expressing cells, the viral genomic RNA level was significantly higher compared with that in the control cells (Fig. 4A). When increasing amounts of IMP1-expressing plasmid were transfected, the viral genomic RNA level increased in an IMP1 dose-dependent manner (Fig. 4B). These results suggested that IMP1 might increase the stability of the viral genomic RNA in the producer cells. To test this possibility, actinomycin D was added to the cells to stop transcription and the viral genomic RNA levels were analyzed at various time points. Indeed, the half-life of the viral genomic RNA in IMP1-expressing cells was significantly prolonged compared with that in the control cells (Fig. 4C). These results established that IMP1 stabilized MLV-luc RNA in the producer cells.

### IMP1 was incorporated into virion particles

**Figure 5:**
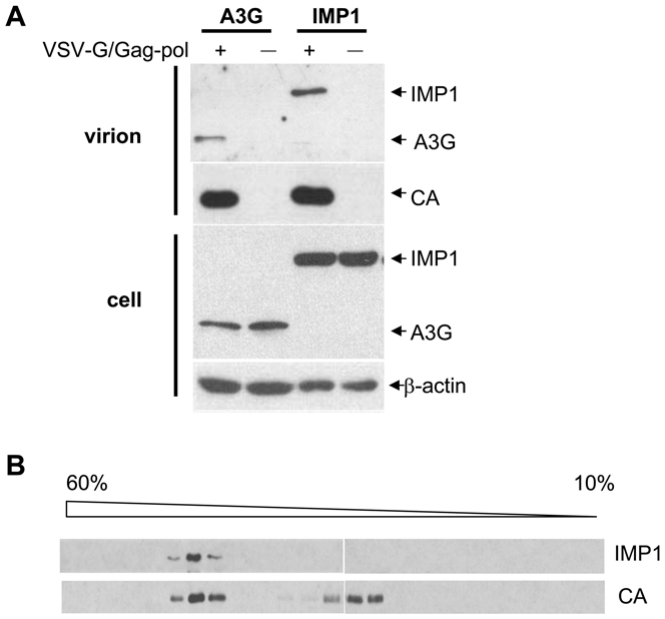
IMP1 was incorporated into the virion particles. Plasmids expressing myc-tagged IMP1 or APOBEC3G (A3G) was cotransfected with the MLV-luc vector producing constructs into 293T cells in 6 cm dishes. At 48 h post transfection, the viruses were collected. (A) The virions were purified first by centrifuging through 25%/45% sucrose step gradient and then centrifuged through 25% sucrose cushion. The indicated proteins in the producer cells and virions were analyzed by Western blotting. (B) The purified viruses were applied to 10–60% linear sucrose gradient centrifugation at 25,000 rpm for 16 h. Fractions were collected, and proteins were precipitated by Trichloroacetic acid (TCA)/actone and analyzed by Western blotting.

We then further analyzed whether IMP1 was incorporated into virion particles. APOBEC3G, which has been shown to be incorporated into virions [34], was used as a positive control. The virions produced from the cells overexpressing IMP1 were harvested, purified by sucrose step gradient centrifugation and analyzed by western blotting. The level of IMP1 was comparable to that of APOBEC3G in the virion particles (Fig. 5A), suggesting that IMP1 was incorporated into the virions. To further substantiate this notion, the virions were subjected to sucrose gradient ultracentrifugation and the distribution patterns of IMP1 and the CA protein were analyzed. As expected, IMP1 co-migrated with CA (Fig. 5B), further indicating that IMP1 was incorporated into the virions.

### Depletion of IMP1 reduced production of infectious MLV-luc vector

**Figure 6:**
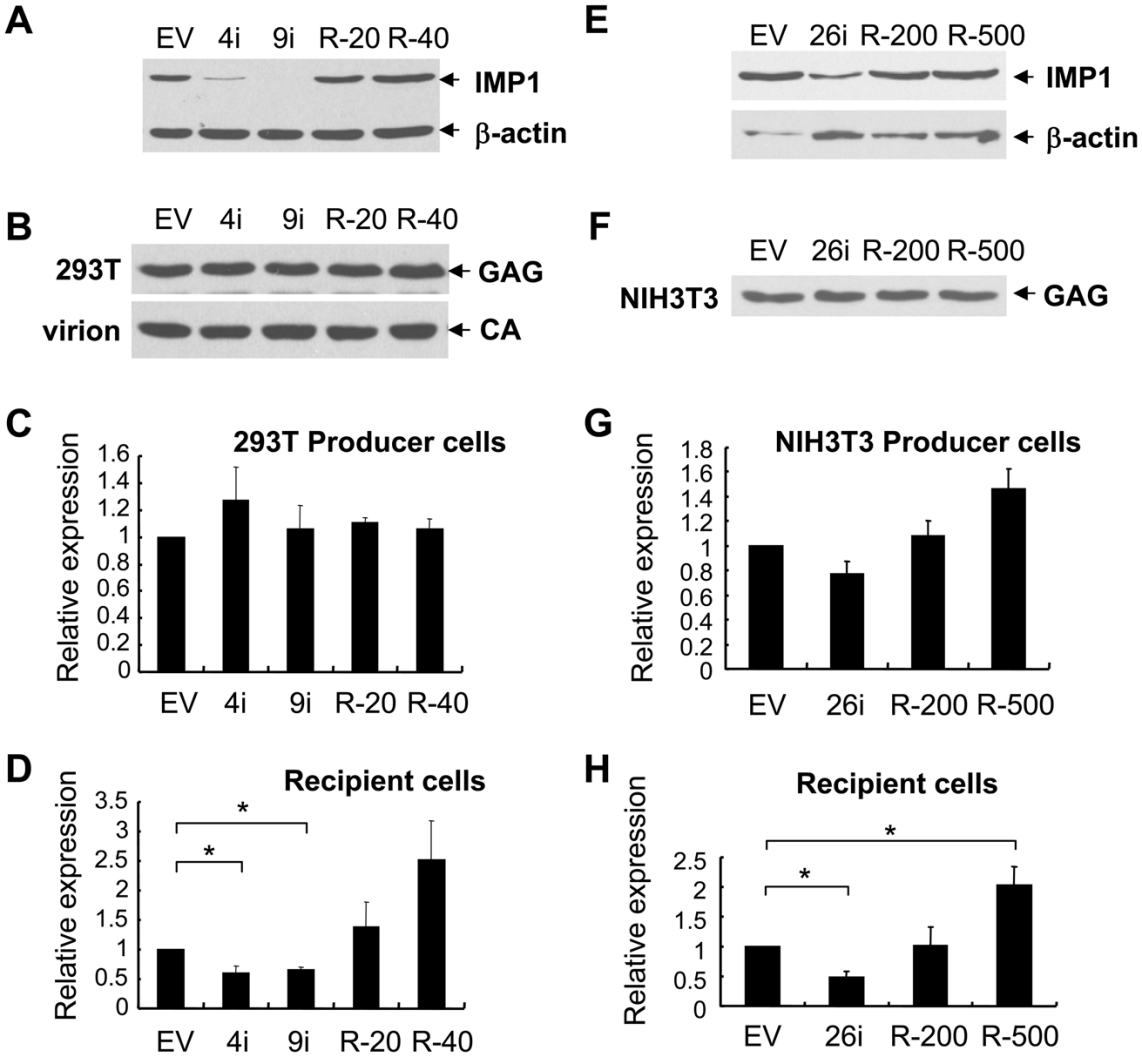
Downregulation of IMP1 in producer cells reduced the production of infectious MLV vector. (A) The plasmid expressing myc-tagged hIMP1 was cotransfected into 293 cells in 3.5 cm dishes with the shRNA directed against hIMP1 alone or with the shRNA plus myc-tagged mIMP1. The expression levels of IMP1-myc were measured by Western blotting using the anti-myc antibodies. 4i and 9i: shRNAs directed against different sequences of hIMP1; R-20: shRNA 9i plus 20 ng of mIMP1-expressing plasmid; R-40: shRNA 9i plus 40 ng of mIMP1-expressing plasmid. (E) The plasmid expressing myc-tagged mIMP1 was cotransfected into 293 cells in 3.5 cm dishes with the shRNA directed against mIMP1 alone or with the shRNA plus myc-tagged hIMP1. The expression levels of IMP1-myc were measured by Western blotting using the anti-myc antibodies. 26i: shRNAs directed against sequence of mIMP1; R-200: shRNA 26i plus 200 ng of hIMP1-expressing plasmid; R-500: shRNA 26i plus 500 ng of hIMP1-expressing plasmid. The plasmids expressing the indicated shRNAs were transfected into 293T cells (B–D) or NIH 3T3 cells (F–H). At 24 h posttransfection, the cells were transfected again with the shRNA-expressing plasmids, together with MLV vector producing constructs and reneilla luciferase expressing plasmid pRL-TK. At 48 h posttransfection, the virus was harvested to infect 293 cells. At the same time, luciferase activities in the producer cells were measured. At 48 h postinfection, the luciferase activity in the recipient cells was measured. (B) (F) The indicated proteins in the producer cells or virions were detected by Western blotting. (C) (G) Firefly luciferase activity normalized by the reneilla luciferase activity in the producer cells. (D) (H) Firefly luciferase activity in the recipient cells normalized by the reneilla luciferase activity in the producer cells. The data are means ± SD of three independent measurements. *denotes p<0.05.

To explore whether endogenous IMP1 regulated the production of retroviral vectors, the expression level of IMP1 was downregulated by RNA interference in the producer cells and the production of MLV-luc vector was assayed. The plasmids expressing shRNAs directed against hIMP1 were first confirmed for their ability to downregulate the expression of hIMP1 in 293 cells (Fig. 6A). MLV-luc producing constructs were cotransfected with the plasmid expressing IMP1 shRNA. The virus was used to infect 293 cells. Downregulation of the endogenous hIMP1 did not affect the expression of Gag in the producer cells (Fig. 6B, upper panel), the protein level of CA in the virions (Fig. 6B, lower panel), or the luciferase activity in the producer cells (Fig. 6C). However, the luciferase activity in the recipient cells was significantly reduced (Fig. 6D). Cotransfection of an IMP1 rescue plasmid removed the inhibitory effect of IMP1 shRNA (Fig. 6D) Similar experiments were also performed in NIH3T3 cells and similar results were observed (Fig. 6E-H). These results indicated that endogenous IMP1 also regulated the production of retroviral vectors.

### IMP1 had little effect on the production of wild-type MLV

**Figure 7:**
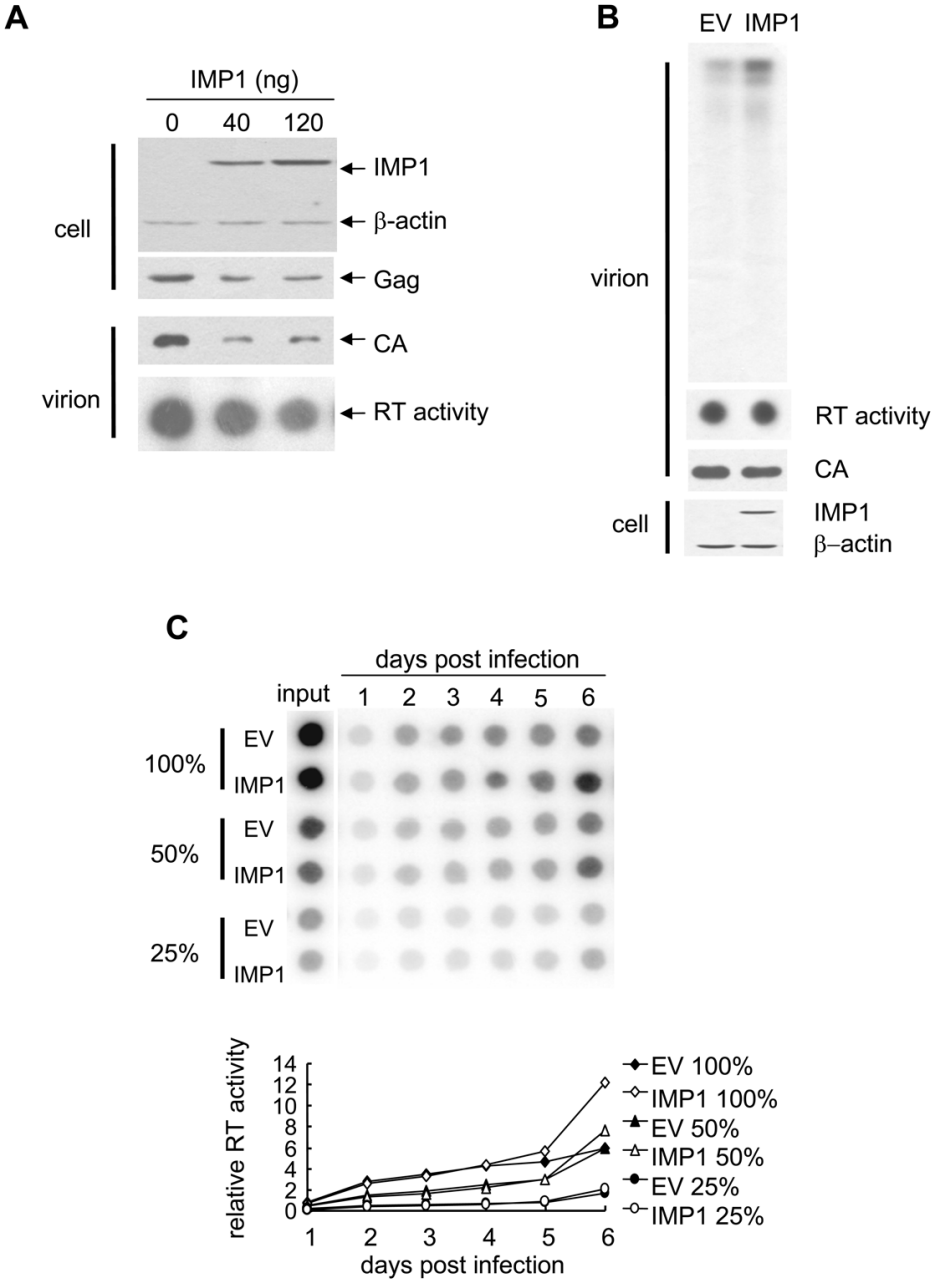
IMP1 had little effect on the production of wild-type MLV. The wild-type MLV producing construct, pNCS, was transfected into 293T cells in 6 cm dishes with the indicated amounts of IMP1-expressing plasmid. (A) At 48 h postinfection, the viral protein levels were measured by Western blotting and the RT activity in the virions was measured by the homopolymer assay. (B) At 48 h posttransfection, the virus produced in the absence or presence of 40 ng IMP1 was collected, purified by 25%/45% step gradient centrifugation and then concentrated by centrifugation through 25% sucrose cushion. Virions containing equal amount of CA, as judged by Western blotting (middle panel), was used to perform the endogenous RT assay. Viral DNA products were detected by PAGE and autoradiograph (upper panel). (C) The indicated amounts of MLV produced in the absence or presence of 40 ng IMP1 were used to infect Rat2 cells. The virus propagation was monitored by measuring the RT activity in the supernatant. Input: the RT activity in the supernatant used to infect Rat2 cells.

To examine whether IMP1 exerts the same effect on the production of wild-type MLV, the virus was produced in the presence of IMP1. Consistent with the above result that IMP1 inhibited the expression of luciferase from the viral genomic RNA (Fig. 1B and E), the protein levels of CA and RT were both reduced in the presence of IMP1 (Fig. 7A). In contrast, the genomic RNA level in the virions, as measured by the endogenous RT assay, was increased in the presence of IMP1 (Fig. 7B). The effect of IMP1 on the production of wild-type MLV was further evaluated by measuring the propagation of the virus produced in the absence or presence of IMP1. To demonstrate that this assay was sensitive enough to detect subtle difference in the amount of the input virus, different amounts of virus was used to infect Rat2 cells. The propagation of the virus was monitored by measuring the RT activity in the supernatants. As expected, IMP1 had little effect on the production of wild-type MLV (Fig. 7C).

## Discussion

Efficient packaging of virial genomic RNA is critical for the production of high-titer retroviral vectors. In this report, we demonstrated that overexpression of IMP1 in the producer cells facilitated the packaging of viral genomic RNA and thereby improved the production of infectious MLV-luc vector in an IMP1 expression level-dependent manner (Fig. 1, 2). Further analyses revealed that IMP1 increased the stability of the viral genomic RNA in the producer cells (Fig. 4). It is believed that the newly synthesized unspliced mRNA of MLV are sorted into two non-equilibrating pools, with one serving as the mRNA for translation and the other as the genomic RNA for packaging [19]–[25], [35]. The luciferase activity expressed form the viral genomic RNA was modestly but consistently inhibited in the producer cells (Fig. 1B). This result suggests that in addition to stabilizing viral genomic RNA, IMP1 may also modulate the distribution of the viral genomic RNA in the two pools. This notion is consistent with the observations that expression of IMP1 increased the abundance of the viral genomic RNA in the producer cells by about 2-fold (Fig. 4A) but increased the RNA level in the virions by about 3.5-fold (Fig. 2D). Whether IMP1 is involved in RNA encapsidation awaits further investigation. Furthermore, since IMP1 bound to the viral genomic RNA (Fig. 3) and was incorporated into the virion particles (Fig. 5), it may also help reverse transcription. This would be consistent with the observations that IMP1 increased the RNA level in the virions by about 3.5-fold (Fig. 2D) but increased the titer of the virus by more than 5-fold (Fig. 2F). Further investigation is needed to explore whether and how IMP1 is involved in reverse transcription.

The stimulatory effect of IMP1 on the production of infectious virion particles was specific only for retroviral vectors. In the retroviral vector producing cells, the viral proteins are expressed from constructs separate from that expressing the viral genomic RNA. Therefore, IMP1 increased the abundance of the viral genomic RNA in the pool for packaging without affecting the expression of the viral proteins. For wild-type MLV, the same unspliced viral RNA serves as both genomic RNA for packaging and mRNA for translation. IMP1 inhibited the expression of the viral proteins while increased the incorporation of viral genomic RNA into the virions (Fig. 7A and B). As a consequence, overexpression of IMP1 had little effect on the production of wild-type MLV (Fig. 7C).

In contrast to the stimulatory effect of IMP1 on MLV vectors, it has been reported that IMP1 bound to HIV-1 RNA but inhibited viral genomic RNA packaging [36]. It seems that how IMP1 binds to the viral genomic RNA also affects the effect of IMP1 on viral genomic RNA packaging. Due to the limited number of the targets of IMP1 so far identified [37]–[39], the consensus IMP1 binding site is not clear yet. It is conceivable that sequence variation in the retroviral vectors may affect the binding ability and thus the stimulatory effect of IMP1 on the production of these vectors. For a particular retroviral vector, experimental test may be needed to evaluate the effect of IMP1 on the production of the vector.

## Materials and Methods

### Plasmids

MLV producing plasmids pCMV-VSVG, pHIT60 and pMA-Luc have been previously described [32], [33]. Wild type MLV producing plasmid pNCS has been previously described [40]. The sequences encoding mIMP1 and mIMP3 were PCR amplified from a mouse cDNA library and cloned into pcDNA4/TO/myc-HisB (Invitrogen). pcDNA4/TO/myc-HisB-APOBEC3G (A3G) was constructed by a cloning the coding sequence of A3G into pcDNA4/TO/myc-HisB such that A3G is tagged with the myc-epitope at the C-terminus. The primers are listed below, with the built-in restriction sites underlined:

IMP1-SP: 5′-CCGGAATTCGCCACCATGAACA AGCTTTACATCGG-3′;

IMP1-AP: 5′-AAGGAAAAAAGCGGCCGCCCTTCCTCCGAGCCTGGGCCA -3′;

IMP3-SP: 5′-CCCAAGCTT GCCACCATGAACAAATTGTACATCGGGAA-3′;

IMP3-AP: 5′ -CCGGAATTCGCCTTCCGCCTTGACTGAGGTG-3′;

To generate the shRNAs directed against IMP1, oligonucleotides were designed and cloned into pSuper following the manufacturer's instruction (OligoEgine). The antisense sequence of the shRNAs directed against mIMP1 and hIMP1 are listed below:

mIMP1-26i: 5′- TTATGAGCCAGGATCTTCAGGG-3′.

hIMP1-4i: 5′- TCTTGCTCTACCTTCTTCA-3′;

hIMP1-9i: 5′-TTTCACGATGACCTGGTCG-3′.

### Cell culture

NIH 3T3 cells (ATCC CRL-1658), Rat2 cells (ATCC CRL-1764), 293 cells (ATCC CRL- 1573) and 293T cells (ATCC CRL-11268) were maintained in DMEM supplemented with 1% antibiotics and 10% fetal bovine serum (Invitrogen).

To produce VSV-G pseudotyped MLV-luc, pCMV-VSV-G, pHIT60 and pMA-Luc were transfected into 293T cells. The reneilla expressing plasmid, pRL-TK (Promega), was included to serve as a control for transfection efficiency and sample handling. At 48 h post transfection, the supernatants were collected, centrifuged at 2,000 rpm for 5 min and filtered through 0.45 µm filters to remove cell debris. To purify and concentrate the virion particles, supernatants were first subjected to 25%/45% sucrose step gradient centrifugation at 25,000 rpm with Hitachi P40ST rotor for 2 h at 4°C and then concentrated by ultracentrifugation with 25% sucrose cushion for 2 h. The pellets were resuspended in TNE buffer (50 mM Tris-HCl pH 7.5, 100 mM NaCl and 1 mM EDTA) and stored at −80°C for future use.

The luciferase activities were measured 48 h after transfection or infection by the dual luciferase assay system following the manufacturer's instruction (Promega).

### Antibodies

The mouse monoclonal antibody 9E10 (Santa Cruz Biotechnology) was used to detect proteins with the myc epitope and the mouse monoclonal antibody M2 (Sigma) was used to detect proteins with the Flag epitope. The N-terminal domain of MLV-CA with a His-tag was bacterially expressed and used to immunize rabbits to generate polyclonal antiserum. The antibody was affinity purified using the recombinant CA protein. The rabbit polyclonal antiserum against MLV-RT was a generous gift from Dr. Stephen Goff of Columbia University, New York, USA.

### RT assays

The homopolymer RT assay and the endogenous RT assay have been previously described [41].

### Detection of the viral DNAs

The assays for detecting the minus strand strong stop DNA and nuclear circular DNA have been previously described [42]–[43].

### Measurement of the viral genomic RNA levels

The virions were lysed in Trizol buffer (Invitrogen). To each sample, 10 µg of total RNA of 293 cells was added to help as well as to serve as a control for sample handling. The RNA was extracted, ethanol precipitated and re-suspended. To remove possible DNA contamination, the RNA was treated with the DNA Free kit (Ambion) followed by ethanol precipitation. The viral genomic RNA level was measured by reverse transcription followed by PCR or realtime-PCR using specific primers for the minus-strand strong stop (MSS) DNA [42]. GAPDH was used as a control using primers GAPDH-FP (5′-TCACTGCCACCCAGAAGACTGTGG-3′) and GAPDH-RP (5′-GGTCCACCACCCTGTTGCT GTAGCC-3′). The PCR conditions for the MSS DNA and GAPDH were 94°C for 30 s, 55°C for 30 s, and 72°C for 30 s for 21 cycles for MSS and 22 cycles for GAPDH. The same conditions and primers were used for realtime-PCR analysis.

To analyze the viral genomic RNA levels in the producer cells, the cells were trypsinized, washed and lysed in the Trizol buffer (Invitrogene). The total RNA was isolated following the manufacturer's instruction. The RNA levels were measured by Northern blotting analysis following the procedure described previously [44].

### Detection of retroviral RNA bound to IMP1

pMA-luc was cotransfected with pcDNA4-IMP1 or empty vector into 293T cells. At 48 h posttransfection, cells were washed extensively with diethyl pyrocarbonate (DEPC)-treated PBS. A quarter of the cell was lysed in the Trizol buffer (Invitrogene) to extract total RNA for analyzing the expression of pMA-luc and GAPDH RNAs. The rest cells were lysed in the lysis buffer (10 mM Tris, pH 7.4, 150 mM NaCl, 0.5% NP-40, 0.01% sodium deoxycholate). An aliquot of the cell lysate was used to examine IMP1 expression by Western blotting. The rest lysate was precleared by incubating with mouse anti-HA antibody and proteins G-sepharose (Pharmaicia) in the presence of RNasin (40 u/ml) at 4°C for 2 h, followed by brief centrifugation. The precleared cell lysate was incubated with new protein G-sepharose beads with or without 3 µl anti-myc antibody at 4°C for 2 h. The beads were washed with the washing buffer (10 mM Tris, pH 7.4, 150 mM NaCl, 0.01% sodium deoxycholate) for seven times. The RNA was extracted with the Trizol buffer, treated with DNase I (Ambion) and analyzed by RT-PCR to detect the viral RNA as described above.
